# Glucocorticoids as an Emerging Pharmacologic Agent for Cardiopulmonary Resuscitation

**DOI:** 10.1007/s10557-014-6547-4

**Published:** 2014-08-28

**Authors:** Giolanda Varvarousi, Antonia Stefaniotou, Dimitrios Varvaroussis, Theodoros Xanthos

**Affiliations:** 1 National and Kapodistrian University of Athens, Medical School, MSc “Cardiopulmonary Resuscitation”, Athens, Greece; 2 Hellenic Society of Cardiopulmonary Resuscitation, Athens, Greece; 3 University of Athens, Medical School, 75 Mikras Asias street, 11527 Athens, Greece; 4 MSc Program Cardiopulmonary Resuscitation, University of Athens, Medical School, 75 Mikras Asias street, 11527 Athens, Greece

**Keywords:** Cardiac arrest, Cardiopulmonary resuscitation, Adrenal insufficiency, Cortisol, Glucocorticoids

## Abstract

Although cardiac arrest (CA) constitutes a major health problem with dismal prognosis, no specific drug therapy has been shown to improve survival to hospital discharge. CA causes adrenal insufficiency which is associated with poor outcome and increased mortality. Adrenal insufficiency may manifest as an inability to increase cortisol secretion during and after cardiopulmonary resuscitation (CPR). Several studies suggest that glucocorticoids during and after CPR seem to confer benefits with respect to return of spontaneous circulation (ROSC) rates and long term survival. They have beneficial hemodynamic effects that may favor their use during CPR and in the early post-resuscitation period. Moreover, they have anti-inflammatory and anti-apoptotic properties that improve organ function by reducing ischemia/reperfusion (I/R) injury. However, glucocorticoid supplementation has shown conflicting results with regard to survival to hospital discharge and neurological outcome. The purpose of this article is to review the pathophysiology of hypothalamic-pituitary-adrenal (HPA) axis during CPR. Furthermore, this article reviews the effects of glucocorticoids use during CRP and the post-resuscitation phase.

## Introduction

Although the initial success rate of cardiopulmonary resuscitation (CPR) and hospital discharge with good neurological condition have increased over the past years, the overall results are however still quite disappointing [1, 2]. The use of vasopressors has long been a mainstay of therapy during CPR [3]. However no specific drug therapy has been shown to improve survival to hospital discharge after cardiac arrest (CA) [4].

Emerging evidence demonstrate that CA could cause CA-related adrenal insufficiency that is highly correlated with poor outcome and increased mortality [5–7]. Moreover, adrenal insufficiency may manifest as an inability to increase cortisol secretion during and after CPR [6, 8]. Glucocorticoids have beneficial hemodynamic and anti-inflammatory effects that may favor their use during CPR and in the early post-resuscitation period. They maintain hemodynamic stability by enhancing vasopressor effects and increasing perfusion pressures during and after CPR [9, 10]. Furthermore, they have anti-inflammatory and anti-apoptotic properties that prevent organ toxicity [11]. However, glucocorticoid treatment with low, medium, and high doses has shown conflicting results with regard to neurological outcome [12].

The purpose of this article is to review the pathophysiology of hypothalamic-pituitary-adrenal (HPA) axis during CPR. Furthermore, this article reviews the effects of glucocorticoids use during CPR and the post-resuscitation phase.

## Physiology of the HPA Axis

Acute stress activates both the sympathetic nervous system to release catecholamines as well as the HPA axis [13]. Stressful signals like hypoxemia and hypotension are integrated by the hypothalamus which in turn increases the release of corticotropin-releasing hormone (CRH). CRH circulates to the anterior pituitary gland, where it stimulates the release of adrenocorticotropin (ACTH). The adrenal cortex is stimulated by ACTH to release glucocorticoid (cortisol) [14]. Arginine vasopressin (ADH) is also a stress hormone, released from the posterior pituitary lobe which stimulates to a lesser degree the release of ACTH from the anterior pituitary lobe [15, 16]. The decrease in cortisol release is the result of a negative feedback inhibition loop, which is triggered by a prolonged elevation of serum cortisol. Utilizing the above-mentioned mechanisms, the body controls the secretion of cortisol by keeping it within relatively narrow limits and it responds to stress with increased secretion of cortisol.

**Fig. 1:**
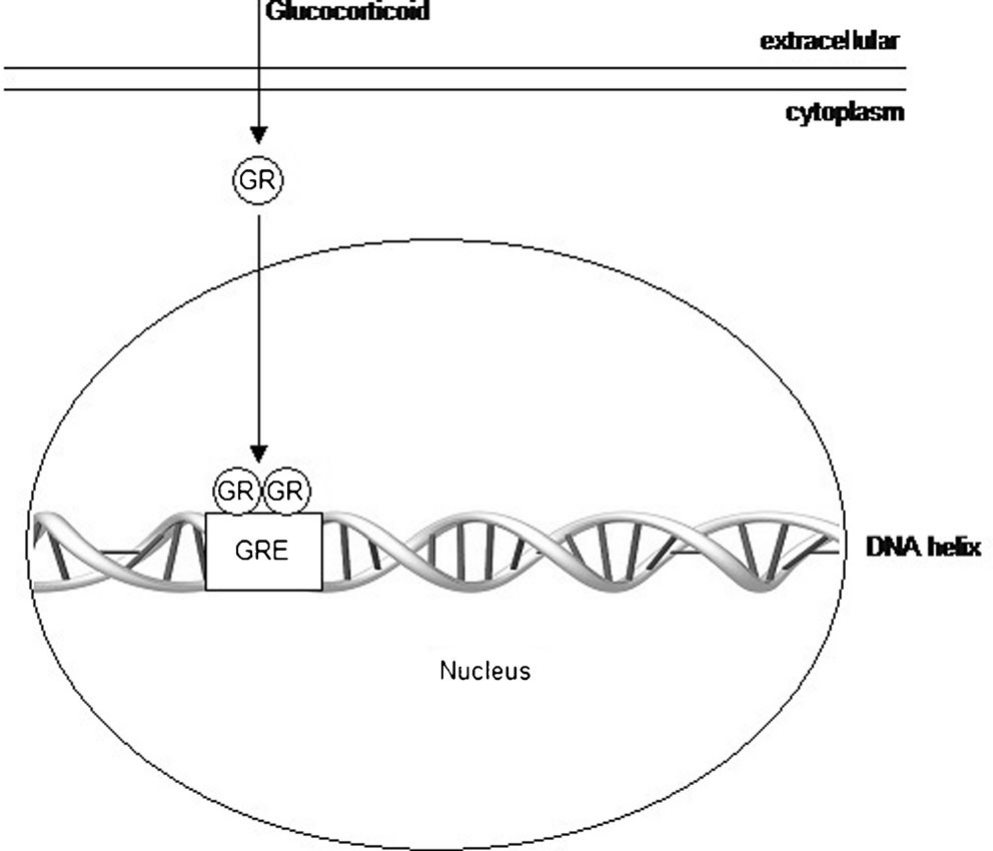
Main mechanism of action of glucocorticoids. Glucocorticoids bind to GR within the cytoplasm of the cell. Upon glucocorticoid binding, the activated GR translocates into the nucleus. GR binds to GRE and affects protein synthesis at the transcription step *GR* glucocorticoid receptor, *GRE* glucocorticoid response element, a stretch of DNA that binds the GR and activates gene transcription

It is known that cortisol is the predominant corticosteroid produced in the human body. Nevertheless, only free cortisol is biologically active; more than 90 % of the circulating cortisol is bound to proteins, predominantly to corticosteroid-binding globulin (CBG), and, to a lesser extent, to albumin [17]. Glucocorticoids exert their effects by binding to and activating an intracellular glucocorticoid receptor (GR) protein (Fig. 1), as well as a mineralocorticoid receptor protein [18, 19]. Intracellular metabolism by 11β-hydroxysteroid dehydrogenase controls the availability of glucocorticoids for binding to the glucocorticoid and mineralocorticoid receptors [19]. Moreover, glucocorticoids may directly interact with cell membranes, as they dissolve into lipid membranes and affect the activity of membrane-associated proteins [19].

## Pathophysiology of the HPA Axis During CA and CPR

Stress imposed by CA results in an acute stress response that is mediated by the activation of the HPA axis. Although an intact HPA axis is demonstrated by increased levels of circulating cortisol, the response of the adrenal gland is inadequate related to the degree of hypoxic stress associated with CA [6]. The no-reflow phase of CA and the low flow of CPR results in inadequate perfusion of the adrenal cortex and impairs the integrity of the HPA axis [20]. The ischemic injury of the adrenal gland leads to adrenal insufficiency, which may be manifested as an inability to increase cortisol secretion during and after CPR. The homeostasis of the neuroendocrine system is damaged, because cortisol is not secreted in response to ACTH, thus resulting in a low concentration of serum cortisol and in high concentrations of the plasma ACTH and ADH. Studies have shown that cortisol levels are relatively low during and after CPR, indicating a dysfunction of the adrenal gland [6, 7]. Schultz et al. pointed out that there was no serum cortisol response at either 6 or 24 h after resuscitation, indicating that the adrenal gland was refractory to ACTH [6]. The low response of the adrenal glands to ACTH in non-survivors indicates a greater suppression of the adrenal function in comparison to the one observed in survivors [7].

Cerebral damage after CA is predominantly located in ‘watershed’ regions of the brain, including the hippocampus [21]. The hippocampus is a key regulatory region for HPA axis negative feedback; therefore, damage from CA/CPR may disrupt negative feedback [22]. Moreover, the HPA axis is extremely sensitive to a cessation of blood flow [23] and studies have shown that prolonged intervals of no flow result in greater ischemic organ injury to the HPA axis. Lindner et al. noted that the interval from collapse to the start of CPR was negatively correlated with serum cortisol concentrations during resuscitation, thus implying an impaired cortisol release with CA [24]. In addition to this, Pene et al. reported that a long interval before initiation of CPR was associated with occurrence of relative adrenal dysfunction [25].

**Fig. 2:**
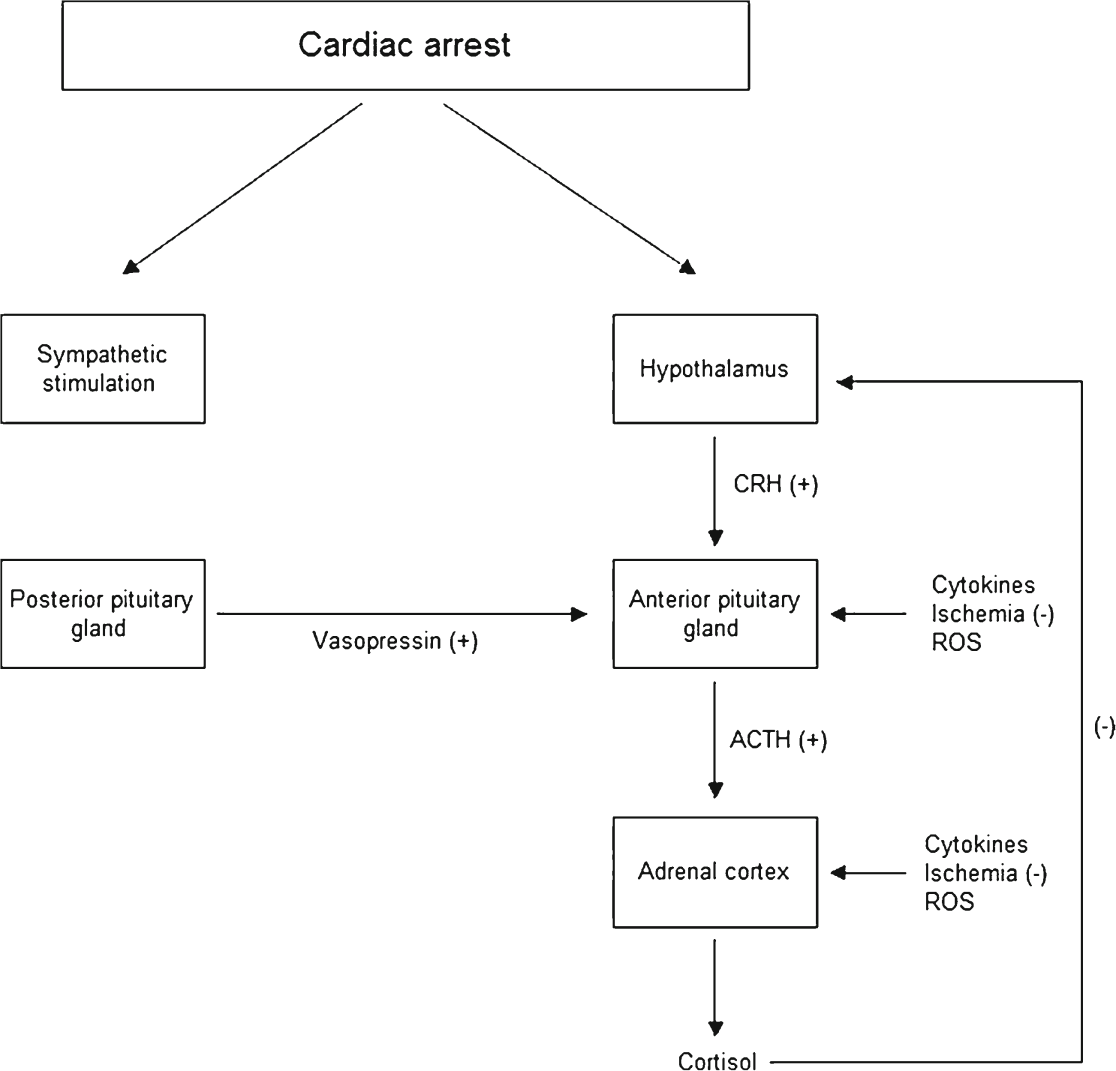
The hypothalamic-pituitary-adrenal axis during cardiac arrest *CRH* corticotropin-releasing hormone, *ACTH* adrenocorticotropin

During CPR and the immediate post-resuscitation phase, pro-inflammatory cytokines (TNF-a, IL-1, IL-6) are released, which dysregulate the axis and lead to adrenal insufficiency as reflected by low cortisol levels [24, 26]. The pituitary response to the hypothalamic corticotropin releasing hormone and the synthesis and release of cortisol from ACTH-stimulated adrenocortical cells is suppressed by the systemic inflammation [27]. Moreover, plasma ACTH becomes biologically ineffective which leads to suppression of the synthesis and release of cortisol from ACTH-stimulated adrenocortical cells (Fig. 2) [20].

## Effect of Serum Cortisol Level on the Outcome of Cardiopulmonary Resuscitation

It is known that stress states are often accompanied by increased circulating cortisol concentrations. Hypercortisolemia in critical illness has been attributed both to stress-induced activation of the HPA axis and to impaired cortisol metabolism [28]. However, in CA ischemic injury of the HPA axis impairs adrenal cortisol release with subsequent decrease in serum cortisol levels. The inadequate response of the HPA axis to the severe stress of CA compared to other stress states makes glucocorticoid supplementation important during CPR. Most studies have shown that adrenal insufficiency may manifest as low serum cortisol concentration during CPR and that serum cortisol levels are lower in non-survivors of CA than in survivors [6, 24, 29]. In a prospective cohort study, Tavakoli et al. measured serum cortisol levels 5 min and 1 h after return of spontaneous circulation (ROSC) in 50 successfully resuscitated patients. They showed that serum cortisol levels were significantly higher in neurologically intact survivors than non-survivors. The authors suggested that serum cortisol levels may serve as a predictor of survival in successfully resuscitated victims of CA [29]. Schultz et al. conducted a prospective study with 205 adult patients presenting with CA and documented an increase in serum cortisol concentrations in survivors during the first 24 h post-ROSC. It was therefore inferred that inadequate cortisol concentrations may play a role in the hemodynamic instability commonly seen after ROSC [6]. It was also suggested that physiologic replacement of glucocorticoids during CPR and during the first 24 h after ROSC may be warranted [6]. Similar findings were demonstrated by Lindner et al.; they reported that during CPR, cortisol concentration was significantly higher in resuscitated patients than in non-resuscitated ones. In addition, according to the above mentioned study no significant correlation between cortisol level and blood pressure in the immediate post-resuscitation phase could be found. However, it is possible that factors that were not determined by the study protocol could have led to this result. Firstly, the study protocol did not allow a precise determination of the relative contribution of post-resuscitation hemodynamic support to the lack of correlation between cortisol level and blood pressure in the post-resuscitation phase. Furthermore, a possible variability of the fraction of protein-bound cortisol, potentially leading to a variable concentration of the biologically active, unbound cortisol, was also not determined [24].

Catecholamines released due to adrenosympathetic discharge induce vasoconstriction and increase vital organ perfusion pressures fascilitating thus ROSC [3]. However, in the cardiac arrest setting, the overwhelming endogenous catecholamine release results in intense vasoconstriction and decreased microvascular blood flow [3]. Moreover, activation of b1-adrenoreceptors increases myocardial oxygen consumption [3]. These adverse effects of catecholamines lead to hemodynamic instability and poor survival outcome [3]. In addition to the action of catecholamines, the stress hormone response during cardiac arrest also includes the release of vasopressin. Vasopressin is a non-adrenergic vasopressor which stimulates the release of ACTH from the anterior pituitary lobe [15] and results in higher adrenal gland blood flow compared with adrenaline, which increases cortisol release [30]. Studies have shown that increased plasma ACTH and cortisol concentrations induced by vasopressin may maintain hemodynamic stability and improve ROSC rate [16, 31]. Kornberg experimented on 14 pigs in order to compare plasma concentrations of ACTH and cortisol, after epinephrine or vasopressin administration in an experimental animal model of CPR. He reported a higher rate of ROSC (100 % vs. 13 %) after the administration of vasopressin than after the administration of epinephrine, despite an almost equal coronary perfusion pressure. The authors reported that the augmented release of cortisol associated with vasopressin may enhance myocardial function during CPR. Although the number of the animals tested was relatively small to lead to a safe conclusion, it is clear that in this study treatment with vasopressin improved survival as well as hemodynamic stability, justifying the need for further research in this area [16]. Additionally, Lindner et al. showed that serum arginine vasopressin and ACTH levels were higher during CPR in successfully resuscitated patients compared to non-resuscitated patients. Moreover, plasma adrenaline and noradrenaline concentrations were significantly higher in patients in whom resuscitation failed than in resuscitated patients, indicating that excessive adrenosympathetic discharge may be associated with poor prognosis after CA [31].

## Actions of Glucocorticoids

Glucocorticoids have metabolic properties. Their various effects include alterations in carbohydrate, lipid, and protein metabolism and maintenance of electrolyte and fluid balance [26]. Glucocorticoids increase blood glucose concentrations and facilitate the delivery of glucose to cells during acute stress [26], increasing the rate of hepatic gluconeogenesis and inhibiting adipose tissue glucose uptake [27]. Glucocorticoids also supply energy to the cell by stimulating free fatty-acid release from adipose tissue and amino-acid release from proteins. In addition, they promote redistribution of body fat and facilitate the effect of adipokinetic agents in eliciting lipolysis of triglycerides in adipose tissue.

Glucocorticoids have a permissive effect on the synthesis of catecholamines and vasoactive peptides [20]. The mechanism postulated includes a cortisol induced inhibition of catechol-O-methyl transferase and a blockade of catecholamine reuptake [32]. Glucocorticoids effects on synthesis of catecholamines and catecholamine receptors are partially responsible for the positive inotropic effects of these hormones [33]. Moreover, they increase systemic vascular resistance [34] and mediate maintenance of peripheral vasomotor tone by facilitating catecholamine-induced vasoconstriction [35]. Glucocorticoids also decrease the production of nitric oxide, a major vasorelaxant and modulator of vascular permeability [36].

Glucocorticoids also have anti-inflammatory and immunosuppressive effects [37]. They attenuate the generation and release of inflammatory cytokines i.e., interleukin [IL-1, IL-3, IL-6, tumor necrosis factor [TNF-a]) [38]. Additionally, they decrease the accumulation and function of macrophages and neutrophils at inflammatory sites [39]. They counteract the acute inflammatory effects on the microcirculation, resulting in vasoconstriction, reduction of edema and a decreased rate of leucocyte adhesion to the injured areas [19].

## Effects of Glucocorticoids Administration During CPR

### Glucocorticoids During CPR in Laboratory Models

Glucocorticoid administration in experimental studies has shown positive results. In a rat model stimulating CA, a dose response investigation of two hydrocortisone dosages (0. 25 mg and 0. 5 mg) compared with normal saline as placebo, showed that 0. 25 mg hydrocortisone was the most effective dose in regard to increasing ROSC rates (*p* < 0. 05). The authors suggest that cortisol improved ROSC by enhancing the cardiovascular effects of epinephrine. However, this study had a major limitation. Rats were anesthetized with pentobarbital prior to CA in order to prevent a hyperadrenal response and it has been acknowledged that pentobarbital induces the liver enzyme system while stimulating steroid clearance, which may have transiently decreased corticosteroid levels in the rats [40]. These results were further supported by a rat model of asphyxial CA treated with methylprednisolone. In this study 20 rats were randomly assigned to 4 treatment groups. The first group received placebo and the second methylprednisolone before CA. The two other groups received placebo and methylprednisolone respectively, after CA. Animals that received methylprednisolone after CA had shorter CPR times required for ROSC although it was not of statistical significance. Moreover, they did not require norepinephrine for vasopressor support and achieved a statistically significant recovery of EEG activity compared to the other animals [41] (Τable 1).

### Clinical Data with Glucocorticoids During Cardiac Arrest

The high mortality rate that has been observed in patients with CA-related adrenal dysfunction and the positive correlation of cortisol levels with survival in CPR raised the question of glucocorticoid supplementation during CPR [6]. The use of glucocorticoids in CPR was first investigated in 1976 with the use of dexamethasone in pulseless electrical activity (PEA). White et al. reported the use of a single intravenous bolus of 100 mg dexamethasone in five patients with PEA. They showed a success in rhythm correction and increase in cardiac output after dexamethasone administration in resuscitation [42]. Moreover, in 1979, White reported that supplementation of corticosteroids in PEA increased ROSC rate and long term survival. However, this study exhibited several limitations as it was a retrospective study with no assessment of CPR quality. Moreover, patients with pseudo-pulseless electrical activity were also likely included in the study [43]. Furthermore, in a small (*n* = 29) prospective, randomized study of patients with pre-hospital bradyasystolic CA, a larger proportion of patients treated with dexamethasone (*n* = 17) were successfully resuscitated and discharged, compared to patients treated with saline (*n* = 12). However, this difference was not statistically significant [44]. Paris et al. conducted a prospective, randomized study with administration of 100 mg dexamethasone in patients with PEA in the pre-hospital setting. They failed to demonstrate any benefit from the administration of dexamethasone in PEA [45]. However, the aforementioned studies demonstrated the effects of glucocorticoids in small sample size studies and did not provide information on the effect of glucocorticoid treatment on CA rhythms other than PEA.

This prospective and non-randomized trial evaluated the effect of hydrocortisone in ROSC rates. In this study hydrocortisone or placebo were given upon patient arrival to the emergency department. The results showed statistically significant increases in ROSC rates with 100 mg hydrocortisone (90 % vs 50 %, *p* = 0. 045). Additionally, serum cortisol levels were not correlated with ROSC rates. Nevertheless, there was no significant difference between the two groups in terms of short-term survival and hospital discharge [10]. These findings are in agreement with previous studies which showed that administration of glucocorticoids improved ROSC rates through supplying adequate serum cortisol levels [24, 29]. Moreover, the authors reported that the incidence of electrolyte disturbances, infection, and GI tract bleeding in the early phase after ROSC did not increase with hydrocortisone use. However this study had several limitations as it was non-randomized and open-labeled and could potentially suffer from selection bias. Moreover, this pilot study had a small sample size and did not assess glucocorticoids efficacy with respect to neurologically favorable survival to hospital discharge [10].

Vasopressin augments the release of cortisol and improves the rate of ROSC compared to epinephrine during CPR in an animal study [16]. Therefore studies investigated the administration of stress hormone “cocktails” in order to improve the outcome of patients in cardiac arrest. In a double-blinded randomized single center study, 100 patients with CA were treated with either the combination of epinephrine and vasopressin during CPR and glucocorticoid supplementation during and after CPR or epinephrine alone during CPR and no steroids. Patients who received the drug combination had more frequent ROSC (81 vs 52 %, *p* = 0. 003) and improved survival to hospital discharge (19 % vs 4 %, *p* = 0. 02) vs patients who received only epinephrine. Furthermore, patients who received hydrocortisone in the post-resuscitation period had improved survival to hospital discharge (30 % vs 0 %, *p* = 0. 02), improved hemodynamics and central venous oxygen saturation compared to patients who received placebo. Moreover, the authors pointed out that glucocorticoid administration increased the efficacy of vasopressors in maintaining adequate perfusion pressures. Glucocorticoids enhanced the inotropic actions of catecholamines and maintained vascular tone [46]. However, this study only refers to in-hospital CA. Out-of-hospital trials must be conducted, given the different natures of these two populations. It must also be taken into account that the issue of chest compression quality was not addressed in this study. Moreover, patients were given a combination of vasopressors with glucocorticoids, which made difficult to isolate the effect of glucocorticoids on the outcome. Thus, it is unclear if these findings were related to corticosteroid supplementation or the combination of the medications used [46].

In a randomized, double-blind, placebo-controlled study conducted by Mentzelopoulos, 268 patients with CA were randomly assigned to receive either epinephrine and vasopressin during the first five CPR cycles (*n* = 130), or epinephrine plus saline placebo (*n* = 138). During the first CPR cycle after randomization patients in the epinephrine, vasopressin, methylprednisolone group received methylprednisolone (40 mg) compared to saline placebo in the control group. Shock after resuscitation was treated with stress-dose hydrocortisone in the epinephrine, vasopressin, methylprednisolone group, (*n* = 76) or with saline placebo (*n* = 73). The combination of vasopressin and epinephrine along with methylprednisolone during CPR and hydrocortisone in post-resuscitation shock, resulted in improved survival to hospital discharge with favorable neurological status, compared with epinephrine and saline placebo (*p* = 0. 02). The authors suggested that methylprednisolone during CPR conferred benefits with respect to hemodynamics by potentiating the vasoconstrictive effects of vasopressors [9].

However, other studies show conflicting results regarding neurological outcome. In a retrospective comparison of low, medium and high steroid dose treatment to placebo in 191 patients with 8 h of ROSC after CA, the outcomes were similar with respect to one year mortality or neurological recovery (*p* = NS). The authors concluded that glucocorticoid treatment was not likely to be beneficial after CA. However, this study had limitations. There were differences between the steroid-treated and the non-steroid treated groups with respect to baseline characteristics and CA etiology potentially favoring the no steroid-group. Moreover, this study evaluated a variable steroid treatment with low, medium, and high doses and not a stress-dose steroid supplementation [12]. Furthermore another non-randomized retrospective study examined the effect of steroid treatment on the outcome of 458 consecutive patients admitted after out-of-hospital CA. In that study patients were assigned to either receive glucocorticoids or saline. No significant differences in survival or neurological recovery were identified [47]. However, the aforementioned studies both exhibit limitations, as they were retrospective and non-randomized. Moreover, there was a variation in the dose of steroids as well as in the duration of steroid treatment. In addition to that, the protocol of the first study [12] included post-resuscitation hyperventilation, which could compromise cerebral perfusion and adversely affect the neurological outcome. Finally, in the second study there was no pre-specified determination of hemodynamic targets [47]. Thus, the above methodological and clinical practice differences could possibly explain the discrepancy in the results regarding the neurological outcome with recent studies [9, 46]. Large, multicenter, randomized, placebo-controlled studies must be conducted in order to further evaluate the effect of glucocorticoid administration in global cerebral ischemia.

Several studies have addressed the relationship between timing of glucocorticoid administration and outcome of CPR. Glucocorticoid administration within 22 min after CA showed a higher ROSC rate in patients with witnessed CA (9). Moreover, in another study glucocorticoid treatment within 8 h after ROSC did not improve neurological recovery. The authors suggest that the negative outcome of CPR was related to late administration of glucocorticoids (12). Furthermore, Paris et al. failed to demonstrate any benefit from the administration of dexamethasone in PEA in the pre-hospital setting. They suggested that further studies with earlier and higher doses of glucocorticoids must be conducted [45]. Mentzelopoulos et al. showed that a combination of vasopressors with glucocorticoid administration during CPR resulted in improved hemodynamics and survival. Although these results cannot be attributed solely to methylprednisolone [46], early CPR drug administration might improve resuscitation outcome, especially in out-of-hospital CA [48, 49]. Further studies must be undertaken in order to clarify whether early administration of glucocorticoids could improve the outcome of patients resuscitated after CA (Τable 1).

**Table 1:** Summary of studies regarding the use of glucocorticoids in the setting of cardiac arrest

Study	Year	Material	Result
Mentzelopoulos et al. [8]	2013	Prospective randomized clinical study	Combined vasopressin- epinephrine and methylprednisolone during CPR and hydrocortisone administration in post-resuscitation period, hydrocortisone in post-resuscitation shock, resulted in improved survival to hospital discharge with favorable neurological status
Tsai et al. [9]	2007	Prospective non randomized clinical study	Hydrocortisone administration during CPR improved ROSC rate
Jastremski et al. [11]	1989	Prospective non-randomized clinical study	Glucocorticoid treatment after ROSC failed to demonstrate a beneficial effect on survival rate and neurological function
Smithline et al. [38]	1993	Rat model of VF-induced CA	Hydrocortisone administered during CPR significantly increased ROSC rate
Katz et al. [39]	1989	Rat model of asphyxia- induced CA	Methylprednisolone given post CA facilitate ROSC and return of EEG activity
White [19]	1976	5 patients with pulseless idioventricular rhythms	Dexamethasone administered during CPR corrected rhythm and increased cardiac output
White et al. [40]	1979	24 patients with pulseless idioventricular rhythms	Dexamethasone administered during CPR increased ROSC rate and long term survival
Schwitzer [41]	1983	Prospective randomized clinical study	Dexamethasone administered during CPR improved initial resuscitation (ROSC) and hospital discharge
Paris et al. [42]	1984	Prospective randomized clinical study	Dexamethasone administration failed to demonstrate a beneficial effect on long term survival
Mentzelopoulos et al. [43]	2009	Prospective randomized clinical study	Combined vasopressin- epinephrine and methylprednisolone during CPR and hydrocortisone administration in post-resuscitation period improved ROSC and survival to hospital discharge
Grafton et al. [44]	1988	Retrospective non-randomized clinical study	Glucocorticoid administration failed to demonstrate a beneficial effect on survival or neurological recovery

*CPR* cardiopulmonary resuscitation, *ROSC* return of spontaneous circulation, *CA* cardiac arrest

## Post-Cardiac Arrest Syndrome

### Glucocorticoids and Post-Resuscitation Myocardial Dysfunction

Post-resuscitation myocardial dysfunction is characterized by impaired contractile function and variable diastolic dysfunction, which resolve within hours or days after ROSC [50]. I/R injury leads to generation of reactive oxygen species (ROS). ROS leads to lipid peroxidation products and oxidatively modified proteins. Free radical-mediated oxidation of membrane phospholipids and proteins is associated with destruction of critical biomembrane structures and is an important mechanism in the pathophysiology of myocardial dysfunction. Glucocorticoids reduce peroxidation of lipids and proteins and stabilize cell membranes [51, 52]. They attenuate oxidative stress and therefore enhance recovery of function in post-ischemic, reperfused myocardium [53].

The release of cytochrome c from mitochondria to cytoplasm plays an important role in the development of I/R injury [54]. Cytochrome c is an essential component of the mitochondrial respiratory chain. It is a soluble protein, attached to the inner mitochondrial membrane [55]. During reperfusion the inner transmembrane potential is decreased and is followed by an increase in the permeability of the outer mitochondrial membrane with subsequent release of cytochrome c from the intermembranous space into the cytoplasm. This mitochondrial permeability transition followed by cytochrome c release may trigger apoptosis [56]. Studies have demonstrated that apoptosis significantly contributes to a reduction of the contractile response of cardiomyotes with reperfusion of ischemic myocardium [57]. Glucocorticoids may reduce the release of cytochrome c from mitochondria and attenuate apoptosis. Moreover, glucocorticoid-induced attenuation of myocardial apoptosis might improve post-ischemic cardiac function [58].

Furthermore, mitochondrial calcium overload during reperfusion is an important mediator of post-resuscitation myocardial dysfunction [50]. Excessive Ca2 influx within mitochondria during I/R injury causes inhibition of mitochondrial ATP production [59] and induces cardiomyocyte apoptosis [60]. In addition, calcium influx during ischemia and reperfusion activates calcium-dependent proteases which are implicated in the degradation of cytoskeletal and contractile proteins in myocytes and therefore compromise ventricular function [61]. Glucocorticoids have the ability to accumulate calcium and preserve calcium homoestasis. Besides, they protect mitochondria from utilizing their high-energy compounds to continuously pump calcium and also allow the resumption of ATP synthesis. In this way they protect hypoxic mitochondria to remain not only ultra-structurally intact, but also functionally intact [62].

Myocardial dysfunction after CA has been attributed to the activation of the inflammatory cascade and to subsequent leukocyte-mediated injury. IL-6 is released during I/R injury and enhances neutrophil-endothelial adherence and subsequent reperfusion injury [63]. The cytokines interleukin IL-1, IL-6, IL-8 and TNF-a, synergistically depress myocardial contractile function [64]. They exert a negative inotropic effect probably mediated by nitric oxide (NO). Studies have shown that IL-6 inhibited myocardial contractility through production of NO, suggesting thus that the production of IL-6 might be a pathogenic factor in the stunned myocardium [65]. Moreover, IL-1 and TNF-a induce down-regulation of adrenergic responsiveness in the myocardium. They inhibit cardiac contractile responsiveness to b-adrenergic stimulation, which may contribute to reversible impairment of cardiac function [66]. Glucocorticoids have anti-inflammatory effects which lead to a repression of pro-inflammatory cytokines and leukocyte adhesion [67]. In that way they enhance the function of the myocardium in the acute setting of myocardial I/R injury [68].

Glucocorticoids may also have positive inotropic and vasodilator properties [43, 69]. By binding to glucocorticoid receptor (GR), they activate endothelial nitric oxide synthase (eNOS), leading to an increased production of nitric oxide. Nitric oxide possesses anti-inflammatory and vasodilatory properties [69]. Vasodilation leads to the reduction of afterload and enhances the recovery of a post-ischemic, reperfused myocardium. Glucocorticoids also have a positive inotropic effect and may enhance the contractile function of myocardium during I/R injury [43]. Moreover, glucocorticoids exert a direct effect on electrical–mechanical coupling in a hypo-perfused myocardium and improve cardiac output [53].

Although the results of experimental studies regarding the cardioprotective role of glucocorticoids are quite encouraging, attention is required at the case of extrapolating the present experimental results to the clinical situation. Factors that affect glucocorticoid treatment like dose, timing and duration of administration, free cortisol level, effect-site concentration, level of downregulation of glucocorticoid receptors, metabolism and clearance rates, and even concurrent treatment may affect the efficacy of glucocorticoid treatment in clinical studies as they cannot be effectively controlled or measured [53, 67, 68]. As a result, this may lead to a discrepancy in results between experimental and clinical studies.

### The Beneficial Role of Glucocorticoids in Systemic Ischemia/Reperfusion Response

The recovery of spontaneous circulation after CA leads to a global systemic I/R syndrome [70]. I/R initiates an acute inflammatory response contributing to post-resuscitation shock [24]. It is characterized by the release of suppressive cytokines (i.e., interleukin IL-1, IL-2, IL-3, IL-6, TNF-a) [38] suppression of the adrenal function and activation of coagulation pathways in the microcirculation [71].

Post-resuscitation shock shares common features with septic shock such as reversible myocardial dysfunction, vasodilatation and plasma cytokine elevation [72]. In the post-resuscitation phase adrenal insufficiency induces hypotension and reduces the effectiveness of vasopressors [73]. These hemodynamic alterations [74] render patients at high risk for acute organ hypo-perfusion and multiorgan system dysfunction [75]. The relationship between adrenal dysfunction and post-resuscitation shock was analyzed in several studies. Kim et al. reported in a prospective study that relative adrenal insufficiency may be associated with increased mortality rate in patients with ROSC [26]. Two other studies reported that low cortisol levels caused organ damage by reperfusion injury [7, 29]. Hekimian et al. reported that patients who die of early refractory shock after CPR may have an inadequate adrenal response [76]. Moreover, Pene et al. assessed the prevalence of relative adrenal insufficiency in patients successfully resuscitated after CA and its prognostic role in I/R syndrome. They showed that the presence of relative adrenal insufficiency was predictive of multiple organ failure mortality. However, the authors failed to demonstrate adrenal dysfunction as a poor prognostic factor in cases of post-resuscitation hemodynamic instability, due to a small sample size [25]. De Jong et al. argued against adrenal insufficiency contributing to a poor outcome. They suggested that post-resuscitation shock after successful CPR was associated with greater activation of the HPA axis and that low serum cortisol levels did not predict mortality. However, methodological differences could possibly explain the discrepancy of the results between this study and the aforementioned studies, as De Jong et al. evaluated free cortisol levels versus total cortisol levels. Moreover, differences in body temperature management may have activated the HPA axis to a different degree. Furthermore, in long-term survivors, the hemodynamic effects of hypothermia per se in the post-resuscitation phase may have counteracted the activation of the HPA axis [77].

Most studies concerning the effects of glucocorticoids were based on septic shock models. Glucocorticoids improved cardiovascular stability reduced catecholamine dosages and shortened the duration of shock in patients with sepsis [72, 78, 79]. Recent prospective randomized trials have shown that treatment with low-dose hydrocortisone significantly reduced mortality and the need for vasopressor therapy in patients with vasopressor-dependent septic shock [80, 81]. Due to the similarities between post-resuscitation shock and septic shock, studies evaluated a potential benefit of glucocorticoid administration for patients with hemodynamic instability following CA [75, 82]. Mentzelopoulos et al. showed increased efficacy of adding vasopressin and methylprednisolone to epinephrine during CPR and treating post-resuscitation shock with stress-dose hydrocortisone. According to this study, patients in the vasopressin-steroids-epinephrine group had more frequent ROSC and attenuated post-resuscitation systemic inflammatory response and organ dysfunction. In addition, it is shown that increased MAP in the post-resuscitation phase induced hemodynamic stability and improved the outcome of post-resuscitation shock [46]. In another study, the combination of glucocorticoids with vasopressors during CPR and glucocorticoids administration in the early post-resuscitation phase improved myocardial function and post-arrest MAP leading to improved survival and neurological outcome [9]. Although there is evidence that glucocorticoids have beneficial effects with regard to post-resuscitation shock, high quality human trials are still lacking. Further studies are needed to investigate the effect of glucocorticoid supplementation in the post-resuscitation period.

### Glucocorticoids in Post-Cardiac Arrest Brain Injury

I/R syndrome leads to free radical formation which is a major component regarding the pathophysiology of post-CA brain injury [83]. Free radical-induced lipid peroxidation reduces ionic pump activity and increases cell membrane permeability. The integrity of sub-cellular brain membranes structure and function is disrupted [84]. Glucocorticoids have beneficial antioxidant and anti-apoptotic properties and reduce free-radical lipid peroxidation [11, 85–87]. They activate the sodium-potassium pump [88] and stabilize cellular membranes exposed to oxidative stress [11]. Studies have shown that glucocorticoids administration during CA preserved the integrity of membranes structure and improved brain function [41]. In that way they prevent irreversible functional and structural damage in brain [89].

Furthermore, the systemic inflammatory response leads to disturbances in cerebral vascular autoregulation. Alterations in capillary permeability induce vasogenic brain edema and aggravate brain injury [90]. Glucocorticoids exert anti-inflammatory effects, inhibit the adhesion of leukocyte to endothelium and therefore protect the endothelial cells [88]. In that way they enhance capillary permeability, maintain the stability of the vascular microcirculation and improve local blood supply [91]. Studies have demonstrated the efficacy of treatment with glucocorticoids in enhancing neurological recovery and ameliorating the extent of brain edema [92].

I/R injury activates pro-inflammatory mediators like IL-6, 8 and TNF-a which contribute to neurological damage [93]. Pro-inflammatory cytokines induce demyelination and recruitment of neutrophils which aggravates brain injury [94, 95]. Glucocorticoids have anti-inflammatory properties which counteract the inflammatory response to reperfusion injury [96]. Studies have shown that glucocorticoid administration inhibit the expression of pro-inflammatory TNF-a [97]. Additionally, in another study glucocorticoids reduced the expression of pro-inflammatory mediators like IL-6, 8 and enhanced brain function [98].

Cerebral perfusion pressure in the early stages of brain injury is determined mainly by mean arterial pressure (MAP) [99]. Studies have shown that hypotension after CA is associated with higher mortality [77, 99]. Also the improved MAP in patients receiving glucocorticoids possibly contributed to improved neurological recovery by attenuating peri-arrest cerebral ischemia. Glucocorticoid enhanced vascular smooth muscle response to vasopressors contributing to increased MAP [9].

However, glucocorticoid supplementation leads to elevated serum glucose levels and may induce neuronal damage [100]. Studies have shown that glucocorticoids exacerbate hypoxic injury to neurons and impair glucose uptake and metabolism in the brain [101]. The neuronal damage in the presence of hyperglycemia is believed to be related to intracellular lactic acidosis leading to an increase in the formation of oxygen free radicals [102]. Studies have shown that elevated blood glucose or lactate levels on admission were predictive of unfavorable neurologic recovery after CA [103]. Moreover, in another study, the relatively higher blood glucose level during the post-resuscitation period was positively correlated with a poor outcome after CA [104]. Furthermore, hyperglycemia enhances neutrophil infiltration in the brain after ischemia. Therefore, hyperglycemia-enhanced inflammatory response to ischemia/reperfusion might contribute to the exacerbation of the ischemic injury [105]. Due to the hyperglycemia- associated toxicity, an adequate glycemic control and the use of intravenous insulin might be warranted, especially in the cases when glucocorticoids are administered in the post-resuscitation period [9].

Conflicting results have been reported in the literature with regard to the neuroprotective effects of glucocorticoids. Glucocorticoids have an impact on both the survival and death of neurons [106]. A systematic review of focal cerebral ischemia failed to show any specific steroid related benefit or adverse effect [107]. Moreover, two other studies reported that glucocorticoids did not improve neurological recovery following CA [12, 47], while experimental studies failed to show any evidence of neuroprotection. The over-activation of glucocorticoid receptors in the hippocampus by glucocorticoids can be detrimental, enhancing the toxic effects of hypoxia and causing apoptosis [108, 109]. There is also a study that has reported an increase in cellular vulnerability to ischemia after glucocorticoid administration [110]. Moreover, an ischemia-evoked rise in glucocorticoid levels may compromise the synaptic neuronal function after hypoxic injury [109]. Furthermore, in another study, glucocorticoid supplementation impaired neuronal integrity and function [111]. Schreiber et al. reported that systemic corticosteroid administration was associated with a transition to delirium in patients with acute lung injury [112]. The heterogeneity in results regarding glucocorticoids as neuroprotective agents could be attributed to differences in ischemia duration, agents, dosing regimens, route of administration and outcome measurements [113]. Further experimental and clinical studies of greater homogeneity must be conducted in order to investigate the role of glucocorticoids in ischemic brain injury.

## Conclusion

Glucocorticoids during and after CPR seem to confer benefits with respect to ROSC rates and long term survival. The efficacy of stress doses of glucocorticoids in reversing postresuscitation shock is well documented. They maintain hemodynamic stability and improve organ function by reducing I/R injury. However, the effects of glucocorticoids in CPR remain controversial, especially in regards to the neurological outcome. Large scale randomized controlled clinical trials must be undertaken in order to further address low dose glucocorticoid efficacy in CA. Moreover, high quality studies should also determine the optimal steroid supplementation doses, as well as the optimal serum steroid levels during CPR and in the post-resuscitation phase.
